# Social Media as a Catalyst for Policy Action and Social Change for Health and Well-Being: Viewpoint

**DOI:** 10.2196/jmir.8508

**Published:** 2018-03-19

**Authors:** Douglas Yeung

**Affiliations:** ^1^ RAND Corporation Santa Monica, CA United States

**Keywords:** social media, health policy, health promotion, health knowledge, attitudes, practice, social change

## Abstract

This viewpoint paper argues that policy interventions can benefit from the continued use of social media analytics, which can serve as an important complement to traditional social science data collection and analysis. Efforts to improve well-being should provide an opportunity to explore these areas more deeply, and encourage the efforts of those conducting national and local data collection on health to incorporate more of these emerging data sources.

Social media remains a relatively untapped source of information to catalyze policy action and social change. However, the diversity of social media platforms and available analysis techniques provides multiple ways to offer insight for policy making and decision making. For instance, social media content can provide timely information about the impact of policy interventions. Social media location information can inform where to deploy resources or disseminate public messaging. Network analysis of social media connections can reveal underserved populations who may be disconnected from public services. Machine learning can help recognize important patterns for disease surveillance or to model population sentiment. To fully realize these potential policy uses, limitations to social media data will need to be overcome, including data reliability and validity, and potential privacy risks.

Traditional data collection may not fully capture the upstream factors and systemic relationships that influence health and well-being. Policy actions and social change efforts, such as the Robert Wood Johnson Foundation’s effort to advance a culture of health, which are intended to drive change in a network of upstream health drivers, will need to incorporate a broad range of behavioral information, such as health attitudes or physical activity levels. Applying innovative techniques to emerging data has the potential to extract insight from unstructured data or fuse disparate sources of data, such as linking health attitudes that are expressed to health behaviors or broader health and well-being outcomes.

## Introduction

Recent efforts to improve health and well-being have looked beyond medicine and health care to consider the influence of a network of upstream factors such as social connectedness, civic engagement, and the physical environment. However, traditional data collection may not fully capture the upstream factors and systemic relationships that could drive positive change in health and health care in the United States [1]. Furthermore, measuring community attitudes and behaviors to inform policy is frequently accomplished with labor-intensive surveys or interviews. Policy actions that are intended to influence broader social change in upstream drivers, such as the Robert Wood Johnson Foundation’s effort to advance a Culture of Health, will need to incorporate a more varied range of behavioral information to inform those actions, when timely or relevant information is otherwise unavailable. Applying innovative techniques to emerging data has the potential to extract insight from unstructured data or fuse disparate sources of data, such as linking health attitudes that are expressed to health behaviors or broader outcomes.

Social media provides an unprecedented opportunity to understand values and expectations about health, and to track healthy behaviors and outcomes in timely ways. Using Web-based applications, people now create and share a wide range of content that may provide richer insight into the value they place on health and well-being for themselves, their friends and family, and the surrounding community. For instance, the amount of discussion (eg, frequency of Twitter mentions) about healthy eating, physical activities, or stress management suggests how much people are thinking about well-being or engaging in health promotion activities [2]. Social media check-ins, that is, when people post their location at a certain place (eg, at a public park or restaurant), can reveal how often people use healthy places.

These behaviors offer insights into how social media can reflect what people consider important, that is, the topics they consider worth discussing or activities they consider worth their participation. Tracking these health-related conversations or actions over time may provide early indicators about important health events or reveal activity patterns that contribute to, or detract from, health and well-being. Policy makers can then use this insight to inform either targeted interventions or broader, longer-term initiatives. Despite a wide range of academic research on how to categorize and mine social media information, social media remains relatively untapped as a source of information to catalyze policy action and advance social change, specifically for health and well-being. Harnessing this potential will require careful consideration to establish validity and reliability, such as addressing bias in either social media usage or analytic techniques.

### Why Is Social Media Well Suited to Support Policy Action and Social Change for Health and Well-Being?

Rapidly refreshed and constantly changing, social media data can help track attitudes and behaviors along multiple paths toward improving health and well-being. Current policy making to advance cultural change in health and well-being has emphasized multisectoral collaborative efforts. For example, in the Culture of Health framework, there is a focus on a dynamic process of improving population well-being, reliant on large amounts of information that updates rapidly and differs by location, as well as by geographic, demographic, and social sectors. However, traditional public policy measurements, such as tracking health policies, community will, or other community or infrastructure level indicators fall short in providing the depth needed on behavioral insights.

Social media data can fit the bill. They are both communal (ie, containing shared ties and social connections) and individualistic (ie, highly granular) and can be broken down to examine behaviors across geography, demographics, or socioeconomic status. This is because although social media is widely used, it is also heavily used by certain segments of the population (eg, youth, minorities) that may be important for issues of health equity. In a Culture of Health, shifting and influencing mindset and expectations about health and well-being is central to social change [3]. Social media could be a particularly valuable tool to capture the mindset and expectations of people in these groups. For instance, minorities may be more likely than other groups to access the Internet and social media primarily through their mobile phones, and these actions could be better tracked to understand health and well-being behaviors [4].

Data from social media can provide insight into whether people exhibit similar views and behaviors around the importance of health and well-being. This is because social media, and big data more broadly, offer a unique type of naturalistic, behavioral data that are a rich source of information on health attitudes and behaviors. For instance, the amount of online discussion (ie, frequent word use) on a given topic may be related to general interest in that topic, particularly when considering text and sentiment analysis. One study of postings on a weight-loss blog suggested that sharing one’s negative emotions, as indicated by the use of sadness words, was linked to greater success in losing weight [5]. Similar explorations of social media as a way to understand health attitudes and behavior (eg, [6-8]) and track health outcomes (eg, [9,10]) further illustrate the potential in exploring social media data to establish their utility for policy uses.

### Social Media Offers Multiple Pathways to Understand Health Attitudes and Behaviors, Key Elements of Cultural Change to Promote Health and Well-Being

There are many examples of health-policy areas to suggest where social media may offer insight or suggest specific policy implications. For instance, the fields of infodemiology and infoveillance explore the use of social media and other Web-based data for public health, such as to predict disease outbreaks [11], explore opinion about smoking among at-risk populations [12], and investigate the impact of environmental factors such as weather on chronic pain [13]. This work has also sought to demonstrate what is possible from a methodological standpoint, such as determining the geographic distribution of Twitter users providing their location information [14], distinguishing between human Twitter users and bots (automated user accounts) [15], or case studies of social media opinion regarding specific medical conditions [16].

As these examples illustrate, the diversity of social media platforms, and of available analysis techniques, provides multiple ways in which social media track policy-relevant indicators. As outlined in Table 1 and the sections that follow, both social media data and related analytic methods contribute to this potential as a data source to understand health attitudes and behavior. For each of these areas, social media data can either complement existing health measures or provide novel ways of measuring behavior change.

**Table 1:** Social media data and methods for health policy action and decision making.

Social media analysis		Health policy use	Example health policy implication
**Data type**			
	Content (text, photos, video)	Crowdsource data for public health surveillance	Use data to more efficiently inform policy interventions
	Location	Build mapping and mobility patterns	Allocate resources to communities in need
	Network connections	Map patterns of social relationships and interactions	Characterize social relationships and communities
**Analytic method**			
	Content analysis	Identify health attitudes and behaviors	Build alternate measures of well-being
	Network analysis	Characterize networks	Identify spread of health behaviors
	Machine learning and algorithms	Predictive analytics	Monitor for early warning about disease outbreaks

### Social Media Content

Policy makers who wish to estimate the impact of a proposed or newly implemented action generally rely on either new data collection or retrospective information from large-population datasets, both of which are laboriously collected and compiled before they are released. Concrete information about policy impact has to be waited for until new data are released, which may delay adjustments or follow-up efforts for months or even years. By contrast, early indications, such as indications from social media, about important health events or trends could provide policy makers with insight to inform targeted and timely interventions.

Social media postings are often spontaneous and frequent. As a result, the content of these postings is timely and can provide up-to-the-moment information. Moreover, people often post on social media from mobile devices, contributing to immediacy and, frequently, location information. Accumulating these frequent postings enables collection of large amounts of collective information that might not otherwise have been available (ie, “crowdsourcing”). Taken together, these data features may be combined with the content in powerful ways, such as for public health surveillance of disease outbreaks [9] or natural disasters [17]. Such analyses can help monitor progress of interventions or relief efforts, or improve situational awareness. A key use for social media may be to improve our understanding of the prevalence or patterns of incidents (eg, disease spread or outbreaks) that may be currently difficult to detect, measure, or quantify. Beyond discrete events, such as disasters or disease outbreaks, social media can be used to track broader trends in chronic stress, preventable hospitalizations, or global burden of disease; for instance, language use on Twitter tracks with rates of coronary heart disease [10]. However, although academic research illustrates potential for social media-based health surveillance, attempts to put this into practice have raised methodological concerns (see a following illustrative example about Google Flu Trends).

Social media may also help improve access to health information and up-to-date measures of patient and consumer experience with care. For instance, take the case of Hello Health, a small primary care practice. Hello Health doctors employ multiple social media tools, including social networks, blogs, and video chat. According to Hello Health’s doctors, such tools can improve communication between health care providers and patients, leading to increased patient engagement and satisfaction [18]. And as patients can use these social media channels to obtain information directly from their health providers, they can also seek information elsewhere online. Social media platforms provide increasingly detailed information, such as in specialized health forums (eg, WebMD, PatientsLikeMe), or on more general sites. Yelp, a Web-based review site, has partnered with ProPublica to provide additional data (eg, wait times, noise levels) to Yelp listings of health care facilities [19]. Data on these communication patterns could be instructive in understanding how to improve patient engagement, health literacy, and access to care.

The immediacy inherent in social media also suggests some potential pitfalls. Social media data are difficult to validate (eg, linking online speech to offline behaviors), and thus may be less accurate than other, more rigorously compiled datasets. In addition, social media’s rapid refresh cycle may encourage policy makers to focus on transitory quick wins or tactical improvements, to the detriment of longer-term, more strategic efforts.

### Location Information

Policy decisions often depend heavily on maintaining an accurate, up-to-date picture of where residents and visitors are located. Location information may inform everyday decision making about when and where to deploy resources or disseminate public messaging. In emergencies such as natural disasters, evacuation efforts may hinge on reliable location information.

Location-based services (eg, mapping) are a key feature of many social media platforms. Consequently, location data from social media may be crucial to providing updated information about shifts in the physical environment that may be unavailable through other means. A proliferation of mapping efforts using social media-based location information (eg, by health departments), often in real time, have begun to explore geographic variations in values, health literacy, or the spread of disease outbreaks. Broad uses for these efforts could include tracking epidemiological patterns (eg, disease outbreaks, clusters, trends), or human mobility patterns that provide broader information than just health or epidemiological patterns (eg, transportation patterns, seasonal or geographic variations).

Real-time location information from social media can be used for more-accurate situational awareness of physical infrastructure, such as public transportation usage or commute times. Other possibilities to measure community investment in healthy environments include traffic data (eg, Waze), public transportation ridership, or usage of healthy alternatives, such as public bicycles. User “check-ins” on services such as Yelp and Foursquare, where users self-report their locations at specific venues such as gyms or public parks, can indicate demand for healthy places or those with opportunities for social connections. Other location-based information may be less dependent on real-time updates but no less useful. Redfin, an online real estate site, provides housing and neighborhood information, such as affordability, walkability, and safety, which may be useful in considering issues of equity. For instance, overlaying changes in home prices or median income with other location data could show the impact on a community of increasing access to health care, transportation, or healthy foods. By providing opportunities to monitor health and safety, social media and other mobile technologies could offer information about how to address stark geographic differences in life expectancy [20].

Using location information from social media may present certain difficulties. A key issue that may affect data representativeness is that only a very small proportion of social media users choose to disclose location information [14]. This may result in biased location data, particularly if there are other differences between those who choose to share location information over social media, and those who do not. There are also privacy considerations to using location data for policy making. Much can be discerned from knowing where people go, potentially including their home, workplace, or typical activities. When designing health interventions or data collection, policy makers should carefully consider how such potentially identifying characteristics are used.

### Network Connections

As population data are collected, communities are often defined, out of necessity, as a simple agglomeration of characteristics made up of its individual members, without accounting for how those people may be connected. Social connections, however, can constitute a subtle form of group analysis, exploring who is similar, who talks with whom, and who may influence others’ actions and attitudes. Thus, while discerning specific relationships among individuals or subgroups may be difficult, relationship and social network information can be extremely powerful.

Violence prevention efforts have compiled network information to identify and then work with individuals who have been most susceptible to perpetrating, and falling victim to, violence in their communities. Similar networks built from social media data could be used to target health messages (eg, high-risk individuals) or efficiently communicate in disaster situations (eg, notifying individuals to evacuate). For years, schools have built “telephone trees,” linking parents to one another so that important information may be disseminated across the network. Social media, with its explicit relationship links, offer policy makers ready-made telephone trees across entire communities. Insight into these networks could be instrumental in how well a community responds to an emergency, providing up-to-date network maps that could reveal members who are not linked into services, and then informing policy decisions around resource allocation to fill gaps.

### Social Media Content Analysis

Social media content, including message text, photos, and videos, can complement traditional attitudinal measures of health attitudes and behaviors. Insight may be extracted from any text from social media postings. For instance, attitudes regarding perceived sense of community, health interdependence, and civic engagement are linked with well-being, and currently measured by self-reported data [21]. Social media content may provide additional ways to measure perceived sense of community. Web-based conversations where people use more first-person plural pronouns (eg, “we,” “us,” “our”) may suggest greater feelings of group cohesion or sense of community (eg, [22,23]). Alternate ways of measuring community well-being beyond economic indicators (eg, Human Development Index) are also possible. Natural language processing techniques that analyze vast amounts of text, such as those generated from social media, can be employed to develop indicators of literacy, such as creativity, language sophistication, or emergence of new forms of language (eg, slang). Similar automated techniques might also analyze nontext content, such as emoticons or emojis. Text-based measures can use analytic techniques to identify key topics of discussion, and then demonstrate varying perceptions of sense of community or civic engagement around them. This could lead to novel ways to measure civic engagement, as social media use may be predictive of voting behavior [24]. These indicators also reveal some intriguing associations with existing socioeconomic measures. For instance, a community’s usage of linguistic markers of community cohesion may be related to its score on the Gini index (a measure of income distribution) [25], suggesting a possible measure of community inequality.

Social media content can improve ways of understanding health needs. Online reviews on Yelp or other sites could also serve as the basis of alternative measures of satisfaction with health providers and utilization of certain types of care (eg, complementary or alternative medicine). For example, text analysis of patient surveys have identified simple keywords, such as “excellent” or "rude,” that are associated with better or worse patient experience, respectively [26]. The extent to which social media sheds light on patient experience depends on the purpose of that social media platform. Sentiment analysis of a Web-based doctor review site (RateMDs) was used to model state-level health quality statistics (eg, mortality rates, patient likelihood to seek follow-up care) [27]. In contrast, a study of tweets directed at specific hospitals showed no association between sentiment expressed in those tweets and traditional survey measures of quality of care in the hospitals [28]. This difference may be due to the fact that people writing on review sites may be motivated to provide more accurate and specific accounts of their experiences, whereas people directing comments toward health care providers may subtly alter their speech, whether it is conscious or not.

Advances in image and video recognition hold promise to capture insight from photos and videos, an ever-increasing portion of social media content. Photos and videos can show when people engage in physical activity, go outdoors, or other health behaviors. For instance, food photos, which are commonly shared, may reveal information about diet. Similarly, photos of red cups commonly used at parties could indicate problematic drinking in college students [29]. Moreover, such alcohol displays could influence others’ attitudes and perceived norms about drinking behavior. With this more-granular picture, interventions could efficiently target influential individuals or groups to modify health behaviors and advance change in the cultural expectations around health and well-being.

Existing measures of subjective well-being also commonly rely on self-reported data. Complementing or replacing self-reported information with naturally occurring text or behavioral measures could improve the measurement of subjective well-being by removing certain biases (at the same time, of course, potentially introducing others). Multiple types of analyses of social media content and structure could be used for various aspects of well-being; for instance, the City of Santa Monica’s Wellbeing Index analyzed Twitter text, embedded Web links, and social networks to collect sentiment and location information [30]. Perhaps the simplest would be to rely on straightforward sentiment analysis. Large-scale data on positive or negative emotions could be used to measure “happiness” (a component of overall well-being), drilled down to observe local or nongeographically-based communities, and validated against existing efforts to measure community happiness using traditional surveys (eg, Bhutan Happiness Index, Gross National Happiness, Organisation for Economic Co-operation and Development Better Life Index).

Importantly, some social media-based indicators have been linked to well-being outcomes. For instance, linguistic analysis of social media text usage of first person plural pronouns (eg, “we,” “us,” “our”) suggests greater sense of community or group cohesion. This indicator has been linked to increased well-being; according to Schwartz and colleagues (2013): “The use of plural personal pronouns such as ‘we’ and ‘our,’ which we take to be proxies for a communal, prosocial orientation are highly correlated with the presence of LS (life satisfaction), whereas ‘I’ and ‘my’ are highly correlated with its absence.” An analysis of sentiment expressed in tweets sent in various London communities found a positive relationship between sentiment and a community’s socioeconomic well-being [31].

Care must be taken when drawing conclusions from what is said and shared over social media. Because social media such as tweets, Facebook posts, or Instagram photos contain information that people actively choose to share, they can provide a rich source of insight toward understanding attitudes and opinions. However, the data from this content are limited to the extent that people choose to present themselves in certain light, selectively and perhaps unconsciously adding or omitting certain content; for instance, social media information about underlying health status may depend on whether people are more likely to post about salient health issues, or health expectations that may differ by individuals. When linking to health outcomes, analyses of social media content should thus consider the context in which those words are used, rather than strictly basing conclusions on simple keyword usage [32]. Finally, the presence of duplicate, commercial, or spam accounts suggests that not all social media content is posted by individuals and therefore may not reflect health-relevant information. Although such content may not directly reflect individuals’ health attitudes or behaviors, it may be relevant nonetheless, for example, tracking youth exposure to unhealthy Web-based advertising.

### Network Analysis

Social media users forge both implicit and explicit connections that can help understand health interdependence, that is, the extent to which people believe their health is dependent on that of their friends or family. Network analysis of social networking platforms can help track how and whether people believe their health can influence, and be influenced by, others with whom they have social relationships. Social media ties can be either two-way (eg, Facebook friends, reciprocal Twitter mentions) or one-way (eg, Twitter follower; Facebook or Instagram likes) relationships. These directed relationships could help determine causality of associations between Web-based behaviors or suggest the directionality in which attitudes and beliefs spread.

Network analysis may be used to examine multiple aspects of social relationships, such as identifying influential people, characterizing specific communities, and the flow of information. Social media and other forms of Web-based data may also afford opportunities to analyze the implicit networks generated by participant interactions. These include, first, increased participation in the so-called “sharing-” or “gig-” economy, that is, technology-enabled service companies that facilitate exchanges between users (eg, Airbnb, Uber, TaskRabbit). Certain sharing economy platforms may be particularly amenable to this, such as home-sharing sites for travel (Airbnb, Couchsurfing) that encourage its users to meet and share experiences. One approach may be to explore partnerships with the online service companies themselves. Airbnb, for instance, actively participates in public policy issues and encourages community as part of its business, for instance, using its data to measure perceived trust and sense of community in cities [33].

Network analysis of social media may also offer new ways to track the existence and quality of cross-sector collaborations and partnerships among health organizations. Social media metadata of connections, such as Twitter mentions and followers, Facebook friends, or LinkedIn connections, can reveal structure and networks of organizational partnerships. These networks can be compared against health outcomes to show where partnerships are effective in improving well-being and also identify areas where new partnerships and collaborations would be fruitful. Network data on organizational partnerships may also explore integration of traditional community resources and health providers (eg, hospitals) with nontraditional community resources that can also influence health. Given the wide range of social media across topics and uses, social media data may also help examine how nonhealth stakeholders can play a role in improving health and well-being. For example, measuring stakeholder support for health promotion could involve tracking social media mentions of health and well-being within a specific domain. This could involve either the communications from key organizations or other stakeholders in these sectors, as well as mentions of their support from other social media users. This could involve insight about support for workplace wellness programs, a community’s attitudes and perceptions of policing efforts, or measures could track online exposure to healthy or unhealthy content (eg, advertising for unhealthy food, alcohol).

Finally, network methods may be employed not only to characterize interpersonal connections, such as among social media users, but as a way to analyze social media content. Semantic network analysis can be used to identify the co-occurrence and relationships among words. This may be useful in determining what broader linguistic patterns and concept mappings reveal about implicit health attitudes, such as whether people think of poor health as an individual responsibility or a societal failure.

### Machine Learning and Algorithms

Too much data can be as much of a problem as insufficient data. When data volumes are overwhelming, it may be impossible to determine relevant data attributes and appropriate metrics. For instance, policy makers who wish to use social media data to learn about a community must first select a social media platform (eg, Twitter vs Facebook) and then determine whether to use tweet-content information, network metadata, location, images, video, or other attributes. Large amounts of data may also make it difficult or even impossible to recognize important patterns in the data, that is, to locate the signal within the noise. And as with any human endeavors, decision makers may unwittingly incorporate their own cognitive biases in interpreting the data.

Data science techniques such as sentiment analysis and machine learning can help make sense of large amounts of information (eg, combining multiple data sets) to support various forms of decision making. Sentiment analysis is a form of natural language processing that seeks to identify attitudes and emotions that are expressed in the text of, for example, social media postings. Research on Twitter data suggests that sentiment (as indicated by word use) in tweets can be used to model life satisfaction [7], happiness [6], and heart disease mortality [10] and health. Sentiment revealed in social media data can also help predict engagement in healthy behaviors, such as health insurance enrollment [8].

A well-known example in applying machine learning techniques for health predictions is Google Flu Trends. Google searches for flu and health-related terms, compared with data of flu-related doctor visits, appeared to provide early detection of influenza outbreaks, as compared with Centers for Disease Control and Prevention’s existing model based on traditional data collection through the public health system [9]. Subsequent research has provided further evidence of the predictive power made possible by combining Google and Centers for Disease Control and Prevention data [34]. The Google Flu Trends example illustrates the potential value from aggregating this kind of preexisting data, and how these approaches may complement existing public health methods. However, there have been debates over the true predictive power and usefulness of using search data for public health surveillance. Suggesting caution in the use of such data, one analysis suggested that the predictive power of Google Flu Trends’s model was significantly overstated [35] but could be useful in conjunction with other flu-tracking data.

Machine learning algorithms attempt to automatically classify or categorize data, such as identifying topics of discussion or objects in images. These algorithms could be used for predictive analytics that support clinical decision making, to determine pricing based on patient or community outcome data, or to contribute to personalized medicine. Social media data may also contribute to predictive analytics that aid in public health responses or planning. For instance, Yelp reviews and Twitter (see the Foodborne Chicago example) have been used to predict foodborne illness outbreaks [36-38]. Building this type of capacity could allow public health agencies to respond more quickly to unfolding public health incidents, preventing them from becoming full-blown crises. The Chicago Department of Health, which runs the Foodborne Chicago website, provides an example of an early warning system that allows people to report possible cases, and analyzes tweets for reports of food poisoning. Models with sufficient precision or specificity could even suggest imminent outbreaks, allowing authorities to respond proactively to prevent the occurrence of such incidents.

Research into Web-based indicators of trauma is common in several other fields and may be borrowed for the health-trauma context. Traumatic life events or other extremely negative experiences that occur in early childhood may be difficult to measure, either because of underreporting due to stigma or lack of ongoing measures (eg, questions about adverse childhood experiences are no longer asked as part of the Behavioral Risk Factor Surveillance System survey). As an example, a vast number of photos are shared online. Image recognition software is already employed to detect and flag traumatic events. For instance, Facebook flags what it considers questionable or inappropriate content (eg, bullying or child pornography), as well as indications of harm (eg, suicide risk) [39]. Tweets containing content indicative of suicide risk factors are correlated with actual suicide rates [40]. These techniques could be repurposed to detect instances of child abuse, sexual assault, or other adverse childhood events.

Although it may be tempting to assume that machine learning and algorithmic techniques offer impartial and equitable analyses of large volumes of data, bias nevertheless creeps into algorithms as well. This bias can involve either invalid assumptions made by those developing the algorithms, or skewed data upon which the algorithms are trained and then applied. Social media measures that do not account for algorithmic biases as well as skewed social media usage may thus inadvertently exclude or underrepresent certain population segments (eg, poor, rural) from policy making consideration. The example of Street Bump illustrates how well-intended uses of new data sources for forward-looking, informed policy decision making could have caused inadvertent harm to certain segments of the population. The City of Boston introduced a mobile phone app, Street Bump, which allowed users to report potholes they encountered. Unexpectedly, more potholes were reported in wealthier areas than in poorer areas. Mobile phone users were presumably more affluent and also tended to drive in affluent areas. Therefore, a simple algorithm that merely allocated resources according to these results would have deepened existing inequities by widening the gap in transportation infrastructure. Poor-quality transportation infrastructure could in turn hinder emergency responders, limit access to preventive health care, or discourage social interactions and community cohesion. In contrast, high-quality transportation infrastructure could increase access for emergency response or preventive health care, encourage social interaction, and in doing so, potentially shrink equity gaps. Informed uses of such data should therefore consider how and whether methods of either data collection or analysis are representative of the populations being served, and what the potential impact on those populations may be.

## Considerations for Future Social Media Analysis

As illustrated above, social media and other emerging data have the potential to contribute broadly to policies focused on health and well-being, as well as inform how social and cultural change may be underway around the importance of these issues. Policy actions intended to improve well-being must act through multiple channels, including improving communities we live in, services we use, and our attitudes toward health and well-being. Achieving a healthier future will require forward-looking methods to draw policy insight from emerging data sources, build cross-sector partnerships, and take full advantage of technological innovation. To bring this vision to reality, health and well-being policy interventions may benefit from the continued use of social media analytics, which can serve as an important complement to traditional social science data collection and analysis. Although researchers and some communities and policy makers have taken note of social media’s utility, its potential is yet to be fully realized. At the same time, traditional sources of survey or administrative data have significant limitations where quantifiable, behavioral data sources such as social media, mobile devices, or other digital outputs can fill gaps. Efforts to improve quality of life should provide an opportunity to explore these areas, and encourage those conducting national and local data collection efforts to incorporate more of these emerging data sources. To illustrate, the Culture of Health Action Framework includes a measure of health-related discourse, based on Twitter data [41]. In addition, several measures of well-being and health equity use national-level survey or administrative data, which social media data could complement. For example, chronic disease burden is currently measured using disability-adjusted life years, a metric that is calculated using a variety of data sources [42]. Disease monitoring from social media data could be employed to provide a more granular picture, such as trends over time, or revealing social networks that may experience greater burden of specific chronic diseases.

Other Web-based data sources may be useful to complement information drawn from social media. Web searches and other information-seeking behaviors offer additional insight into people’s concerns. In the 2014 contaminated-water crisis in Flint, Michigan, residents began searching for information about contaminated water and, as news coverage continued, broadened their searches to explore potential health impact [43]. Web search activity for health information and resources could be used to measure health attitudes in different communities. Accordingly, search data can be aggregated and then compared with other health data (eg, traditional health surveys or estimates) to build predictive models of large-scale, population-level health and health behaviors. For instance, cancer-related searches are associated with American Cancer Society estimates of cancer incidence and mortality [44].

Going forward, the value of social media data to guide policy making may rest on the ability to continually shape analyses to match the ever-shifting data sources and platforms. In other words, as the nature of social media itself changes, analysis of social media will need to continually evolve. Many important sources of data may not yet exist. Therefore, while analyzing social media data offers many advantages, several key implications should also be considered.

### Limitations

The diversity of social media data sources and analytic methods suggests the need to ensure their validity: that social media data reflect real-world outcomes. Questions remain, for instance, about how to properly interpret what is shared online. What people choose to share may constitute a form of self-presentation or performance to a specific audience, rather than convey their true feelings [45]. Other data validity concerns relate to the commercial nature of social media. Profit-driven incentives may lead social media platforms to highlight certain types of sharing, or Internet service providers to prioritize certain types of Web traffic (ie, “net neutrality”).

To overcome such limitations of social media data, there is a need for validation research. Validation of data and methods may include determining whether measures based on social media data track with other, more traditional measures of the same concept (eg, surveys of attitudes, public health disease monitoring). Another way to validate social media data may seek to relate it directly to real-world behaviors (eg, civic participation, health provider visits, insurance enrollment, organizational partnerships). For instance, one intriguing study showed that Google searches for mental health information follow consistent seasonal patterns, uncovering a potentially useful finding, but one which the authors noted would have to be validated against clinical or other surveillance data [46]. Although other social media research has tried to establish these relationships, much of this work is specific to a data source such as Twitter [47] or Google searches [48]. Establishing broad validity across social media platforms or analytic techniques could help move social media from research to action. Building awareness of and trust in emerging data sources increase the likelihood that they will be used to inform health policy making. Alternately, policy makers may need to accept trade-offs in using social media analytics with low validity for exploratory purposes or in surveillance and monitoring, but not necessarily when accuracy is critical, such as equitably allocating public resources.

The extent to which social media data are representative of either the general population or specific subpopulations suggests another important consideration. Care must be taken so that actions informed by nonrepresentative data do not exacerbate existing inequities by affording certain groups fewer opportunities to be heard, in effect, writing some people out of future narratives and resulting actions. For instance, compared with the general population, social media users tend to be young, educated, wealthy, and living in cities [49]. Ownership of mobile devices, from which social media content is frequently posted and accessed, is widespread but also limited to those who can afford them. People in certain geographic regions may be more likely to use particular social media platforms; for instance, homegrown services such as Weibo and WeChat are primarily used in Asia. Social media measures that do not account for these skewed demographics may thus inadvertently exclude or underrepresent certain population segments (eg, poor, rural) from analyses based on which data source or analysis method is chosen. This could significantly harm those populations if, for instance, public health resources are misallocated away from those in need, or public health interventions ineffectively target health conditions.

However, the demographics of social media users may also offer the tantalizing possibility of reaching population segments that may have been difficult to reach by traditional means of data collection [50]. For instance, samples of Twitter users may disproportionately contain individuals such as African Americans, highly educated and high-income people, and younger and urban-dwelling people [51,48]. Therefore, social media may represent a new opportunity to fully capture the voices and civic engagement of those who may have been marginalized in civic decision making. Future policy measures should seek to advance these important uses for social media and other emerging data.

Finally, one of the most widely discussed implications of social media, “big data,” and other data sources is the potential impact on privacy. For instance, social media users typically do not explicitly provide consent for how their content is eventually used, unlike with traditional data collection (eg, interviews, surveys). Another consideration is that health data, like most other forms of digital data, are most commonly shared on platforms owned by private companies, which may then share that data with other entities [52]. Although individuals may assume that data shared anonymously remains as such, in fact it may be shared widely and even reidentified to infer individual identities or identify sensitive health characteristics. These privacy considerations merit further discussion to determine how to balance public good as well as benefits to individual users against these potential invasions of privacy.

### Moving From Research to Action: Social Media for Policy Actions and Social Change to Advance Health and Well-Being

Social media data can form the basis of policy indicators across multiple health domains. Exploring Web-based discussion of health promotion, for instance, should provide insight into public opinion about health, and can be expected to track broad trends in attitudes and norms about health and inform interventions to shift them. Emerging social media tools and platforms could be used to characterize aspects of well-being that are not currently tracked broadly, but for which a substantial amount of research is available on how to define and measure them, such as social isolation and perceived loneliness, belongingness, toxic stress, and spirituality.

By now, there is a large and diverse body of research suggesting how to extract public health insight from social media data. Translating this knowledge into practice could begin by using social media data and analytic methods to complement existing policy data and practice, or by helping to establish relationships among diverse stakeholders who could work across sectors to advance health and well-being. First, an initial step toward using social media data in practice (eg, research, policy making) could be as a complement to more-traditional data sources that are already in use. Much of the research described in this article has compared information gleaned from social media against that of existing data, or has sought to use social media data as a way to model real-world outcomes, such as health behaviors, social connections, or spread of disease. Decision makers in either the private or public sectors could mine these techniques and topics to determine whether they may be applicable for their needs. Social media data can also be used to provide complementary evidence or analysis to support human decision making, such as individual decision making about health choices or civic decision making about resource allocation and investment strategy. Combining social media and other digital data may also help identify and suggest collaborations or partnerships that yield improved health outcomes. Possible constraints, however, include whether organizations would be willing to share sensitive or proprietary information. Analytic techniques, such as from Fiscalnote, a firm that tracks and attempts to predict legislation, could be used to help identify potential actions or policies to support cross-sector collaborations.

Second, given the commercial nature of most social media data, working with these data can produce fruitful cross-sector partnerships. A collaboration between Yelp, an online review site, and ProPublica is a good illustration of the potential for how public and private sectors can work together to provide consumers with useful health information over social media. Yelp also partnered with the cities of San Francisco and New York, with support from the White House, to provide city data of restaurant hygiene scores on Yelp business pages [53]. Part of this work included creating an open data standard that would allow other interested cities to do the same.

As social media analysis becomes more established in policy making, it will be crucial to ensure data reliability and validity. For example, developing standardized metrics could help assess effectiveness of policies based on social media data and compare policy initiatives, thus facilitating translation of knowledge (eg, infodemiology) into practice [11]. Efforts to improve data reliability could also benefit from using techniques to distinguish between insightful content generated by human social media users and automated posts from bots [15].

Future efforts should also carefully consider how to preserve civil liberties, such as whether machine-learning algorithms introduce inadvertent biases, and how to deal with privacy concerns, such as how health information or personally identifiable information may be used. As described earlier, this is particularly important because a great deal of online health information is held by private corporations [52]. Libert also points out the possibility that people are treated differently or discriminated against on the basis of perceived health information about them, whether or not it is accurate. The implications of how these data are used, either by the companies who hold it or to whom they provide it, warrant further consideration. For instance, what are the conditions under which private corporations will provide or allow access to these data? Should policy makers seek to incentivize partnerships or to prevent issues inherent in-data mining, such as inadvertent discrimination or privacy invasions? Similar privacy concerns also arise in the context of how governments may choose to use health-related insights generated from social media data. Access to vast amounts of social media data could inform policy making to improve health and well-being but could also be misused in ways that undermine health privacy and confidentiality.

In summary, a number of potential approaches could improve the accessibility and utility of social media in policy making. Given that much of these data are held commercially, the vast market opportunities that companies continue to envision with digital health data, as well as increased corporate interest in well-being and social impact, opportunities should exist to partner broadly with stakeholders across a range of sectors. Accordingly, increased global interest in well-being measurement for policy making affords additional opportunities to collaborate and build data analysis capabilities. Taken together, social media and other emerging data sources provide multiple avenues to help track and motivate wide-ranging and truly inclusive policy action to improve health and well-being for all.

## Abbreviations

GDP: gross domestic product
